# Regulating role of abscisic acid on cadmium enrichment in ramie (*Boehmeria nivea* L.)

**DOI:** 10.1038/s41598-021-00322-6

**Published:** 2021-11-11

**Authors:** Kunmei Chen, Ping Chen, Xiaojun Qiu, Jikang Chen, Gang Gao, Xiaofei Wang, Aiguo Zhu, Chunming Yu

**Affiliations:** Institute of Bast Fiber Crops, Chinese Academy of Agricultural Sciences, Changsha, China

**Keywords:** Environmental sciences, Abiotic

## Abstract

Abscisic acid (ABA) is known as an important hormone regulating plant stress resistance, such as salt, drought and heavy metal resistance. However, the relationship between ABA and cadmium (Cd) enrichment in ramie (*Boehmeria nivea* L.) is still unclear to date. This study aimed to reveal the effect of ABA on Cd enrichment in ramie, and we received the following results: (1) Under Cd treatment, the Cd uptake of ramie increased with the increase of Cd concentration, but the chlorophyll content decreased. Under Cd treatment, the ABA content was highest in roots of ramie, followed by that in old leaves, and lowest in new leaves. Long-time treatment of high Cd concentration reduced the ability of endogenous ABA biosynthesis. (2) Spraying ABA on ramie plants (SORP) and adding ABA directly to the culture solution (ADCS) with low concentration can promote the growth of ramie and increase the amount of Cd uptake, and the effect of SORP is better. (3) The molecular reason for the decrease of chlorophyll content due to Cd stress, may be resulted from the down-regulated expression of the chlorophyll synthesis genes (*BnPAO* and *BnNYC1*) and the up-regulated expression of the chlorophyll degradation genes (*BnCHLH*, *BnCHLG*, *BnHAP3A* and *BnPPR1*). The elevated ABA content in ramie plants may due to the up-regulated expression of the ABA synthesis related genes (*BnABA1*, *BnNCED3*, and *BnNCED5*) and the genes (*BnABCG40*, *BnNFXL2*, *BnPYL9*, *BnGCR2*, *BnGTG1*, *BnBGLU1*, *BnUTG1*, *BnVHAG1* and *BnABI5*) that encoding ABA transport and response proteins, which was consistent with the enhance the Cd uptake in ramie. Our study revealed the relationship between ABA and Cd uptake in ramie, which provided a reference for improving the enrichment of Cd in ramie.

## Introduction

Remediation of heavy metals contaminated soil has become an increasingly active field in environmental science research, and also a worldwide problem^1^. Phytoremediation or bioremediation is a process that removing the pollutants from contaminated soil using particular plants of a special absorption and enrichment capacity of heavy metals; the plants are finally harvested and treated with ash, so as to achieve the purpose of controlling pollution and ecological restoration^2^. Phytoremediation is a cost-effective, environmentally friendly, aesthetically pleasing approach most suitable for developing countries^3^. However, there are also problems and difficulties in phytoremediation^4^. The species of hyperaccumulator are few; the growth of most hyperconcentration plants is slow; most of the hyperconcentration plants have been found to be selective to heavy metals and have a good enrichment effect on a single heavy metal. For instance, most of the ferns show strong ability of absorbing and enriching in arsenic, but no hyperaccumulating effect on other heavy metals^5^. In addition, the hyperaccumulators discovered at present show inhibition in growth when planted in polluted areas with poor environment or arid and barren soil, which greatly reducing the effect of repair^6^.

Ramie, (*Boehmeria nivea* L.), family Urticaceae, is a plant native to eastern Asia, and one of the world's oldest fiber crops. Ramie has a well-developed root system, rapid growth, strong reproductive capacity, and high biomass production. The annual dry matter can reach as high as 42 t per hectare per year^7^, which has advantages in removing of heavy metal in contaminated soil. Ramie has strong resistance to adversity: it can still grow well in the fertile and barren mountain area, the extremely arid soil or heavy metal polluted mining area, which makes ramie a good material for the repair of heavy metals contaminated soil^8–10^. Ramie has high tolerance and high absorption to a variety of heavy metals such as As, Cd, Sb, Pb, Mn and others, which makes it advantageous to the remediation of multiple heavy metals^11,12^. In many respects, the biological advantages of ramie have made up for the shortage of the existing superaccumulative plants, such as dwarf plant, slow growth, greatly affected by climate, and difficulty in realizing the practical application value in phytoremediation. Thus, using ramie as repair plants has good ecological benefits. Heavy metals that absorbed by ramie is mainly distributed in the root, few in the fiber^13,14^. The heavy metals absorbed by fiber will be removed in the process of fiber degumming, and it does not affect the quality of fiber. The most important thing is that the heavy metal absorbed by ramie does not enter the food chain and does not pose a threat to the health of animal and human. Therefore, ramie is a pioneer material for phytoremediation of heavy metal contaminated soil.

ABA is known as stress hormone and plays an important role in plant stress physiology^15^. Generally speaking, abiotic stress such as drought, cold, high temperature, salt, waterlogging, and heavy metal stress can increase the content of endogenous ABA in plants and enhance stress resistance. Monni reported that the concentration of endogenous ABA increases with the increase of heavy metal pollution in Finland^16^. Cd stress in the external environment can regulate the synthesis and accumulation of endogenous ABA, and further play the role of resistance to stress. For examples, Cd short time treatment of tomato tubers can increase endogenous abscisic acid concentration^17^; In rice^18^, *Typha latifolia*, *Phragmites australis* and citrus^19^, Cd treatment can also increase the content of endogenous abscisic acid. The stimulation of Cd stress increases the endogenous ABA content of soybean seedlings, thereby enhancing the resistance of soybean seedlings to adversity^20^. The regulation of endogenous ABA synthesis under Cd stress is related to Cd tolerance of rice seedlings. For example, higher ABA content is observed in the Cd tolerance rice strain than that of Cd sensitive strain under the same Cd stress^21^. Exogenous ABA can regulate the Cd accumulation and transport coefficient characteristics of wheat, and can also regulate the damage of Cd stress to plants^22^. Under Cd stress, ABA regulates the growth of rice roots by regulating the accumulation and distribution of auxin in rice, and increasing the expression of genes on auxin and MAPK signaling pathway and cell cycle^23^. In phytoremediation, proper application of ABA can enhance Cd uptake in *Solanum nigrum*^24^. It is obvious that the regulation role of ABA in Cd uptake in plants and the application of ABA to improve the efficiency of remediation of heavy metal contaminated soil in agriculture have become one of the hot topics in plant remediation. However, researches about what is the relationship between endogenous ABA content and Cd absorption capacity in ramie, how to respond to Cd stress in the endogenous ABA of ramie plants, and how to regulate the Cd absorption characteristics of ramie by endogenous and exogenous ABA have not been reported.

In order to explore the role of ABA in response to Cd stress and in regulation of Cd uptake in ramie, the ramie variety Zhongzhu No. 2 of high Cd enrichment ability was used as material in this study.The pattern of endogenous ABA content of ramie plant under Cd stress and the effects of ABA treatment on Cd absorption in ramie were analyzed.

## Materials and methods

### Ethics statement

The ramie variety Zhongzhu NO.2 used in this study was bred by the Institute of Bast Fiber Crops, Chinese Academy of Agricultural Sciences, China. Therefore, no specific permissions were required for using these specimens. All methods comply with relevant institutional, national, and international guidelines and legislation.

### Plant materials

The ramie variety Zhongzhu NO.2, which demonstrates strong Cd tolerance (data unpublished) and is widely cultivated in the south of China, was used in the present study. Cottage seedlings about 15 cm in length were planted in a hydroponic device and cultured in 1/4 Hoagland nutrient solution under the following conditions: 300 μmol m^-2^ s^−1^ light, 12 h light/12 h dark period, 26 °C, and 70% relative humidity. The nutrient solution was renewed every five days.

### Cd stress experiment

When the length of root was 10 cm, plants were treated with 0 (control), 5, 10, 15, and 30 mg L^−1^ CdCl_2_. After 0, 3, 10, 20, and 32 days of treatment, new leaves (the fourth fully expanded leaf counted from the top of the plant), old leaves (the second leaf counted from the bottom of the plant) and roots were separately harvested for ABA content determination. Meanwhile, the fifth fully expanded leaf counted from the top of the plant (Fig. S1) was sampled for quantitative PCR (q-PCR). In addition, three independent biological replicates were established for the control and Cd treatment. Each replicate consisted of 10 ramie plants.

After 25 days of treatment, the top fourth leaf was used for chlorophyll content determination. The fresh leaves (0.01–0.02 g) were collected immediately soaked in 95% ethanol, under dark conditions. The measurement and calculation of chlorophyll content was performed according to the methods described by Sales et al.^25^.

### ABA treatment experiment

When the length of root was 10 cm, plants were treated with 15 mg L^−1^ CdCl_2_. After 4 days of Cd treatment, plants were treated with 0 (control), 0.01, 0.05, 1, 2.5, 5, 20, and 40 μM ABA by two ways—adding ABA directly to the culture solution (ADCS) and spraying on the leaves. After 10 days of ABA treatment, plant height and root length were measured. The increase of plant height and root length was calculated by the formula: 100·(A_10_ − A_0_)/A_10_, A_0_ and A_10_ denote the value of plant height or root length after 0 and 10 days of ABA treatment, respectively. At the 20th day of ABA treatment, leaves of each treatment were respectively harvested for Cd content determination. Meanwhile, leaves (the fifth fully expanded leaf counted from the top of the plant) and roots were sampled for q-PCR. Three independent biological replicates were established for the control and Cd stress treatment. Each replicate consisted of 10 ramie plants.

### ABA content determination

For ABA content determination, about 0.5 g fresh leaves was ground into a homogenate with 9 times (w/v) of 0.1 mol/L phosphate buffer solution (0.1 mol/L Na_2_HPO_4_, 0.1 mol/L NaH_2_PO_4_ and 0.9% w/v NaCl at pH 7.4) in a mortar on ice. The homogenate was then transferred to a 1.5-mL Eppendorf tube and centrifuged at 3500 rpm for 10 min at 4 ℃. Forty μL of the supernatant was collected in a new tube and assayed for ABA content using a plant ABA Elisa kit (NJJCBIO, China) according to the manufacturer’s instructions.

### Cd content determination

For Cd content determination, plants were dried and used to estimate Cd content using a SOLAAR M6atomic absorption spectrometer (Thermo Fisher Scientific, MA, USA) according to the methods described by She et al.^26^.

### q-PCR

For q-PCR, total RNA was extracted from plant samples using a plant RNA purification kit (Tiangen, Beijing, China). Briefly, 0.5 μg total RNA was used to synthesize cDNA (in 10-mL reaction volumes) using a PrimeScript RT perfect real-time reagent kit (TaKaRa, Japan), and the cDNA was subsequently diluted four times. q-PCR was performed using the PC33-2 × Sybr Green qPCR Mix PC3302 (Aidlab, Beijing, China) with the special primers (Supplemental Table). The *18S* rRNA gene (primer-F: TGACGGAGAATTAGGGTTCGA, primer-R: CCGTGTCAGGATTGGGTAATTT) was used as the internal control to normalize q-PCR data. q-PCRs were performed in the Bio-RAD CFX96 (Bio-RAD) according to the manufacturer’s instructions. Expression of target genes was defined from the threshold cycle, and relative expression levels were calculated using the 2^−ΔΔCt^ method, after normalization based on the reference gene.

### Statistical analysis

Calculations were performed in Excel 2010 (Microsoft, Redmond, Washington, USA), and the results are presented as means ± standard deviation (SD). Statistical analysis was conducted using one-way analysis of variance (ANOVA) with SPSS Statistics 19.0 (SPSS Inc., Chicago, IL, USA). Comparisons of means were performed using the least significant difference (LSD) test at *P* = 0.05. A difference between means was considered statistically significant when *P* < 0.05.

## Results

### Changes of ABA content in ramie plants under Cd stress

**Figure 1:**
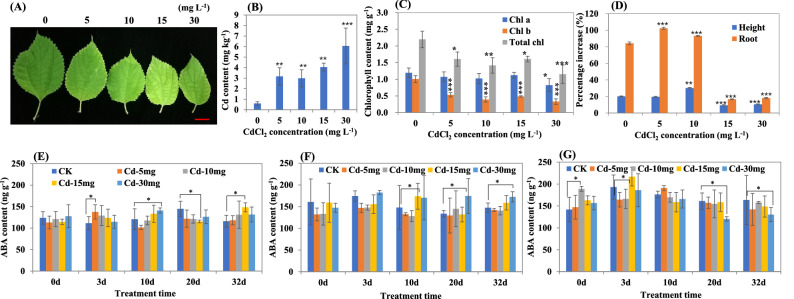
Phenotype and ABA content of ramie plants under Cd stress. (**A**) Phenotype of leaves under various concentration of Cd treatment. The red bar denotes 2 cm in length. (**B**) Cd content of shoots under Cd stress. (**C**) Chlorophyll content of the top fourth leaf under Cd stress. Data are means ± SD of three biological replicates. Each replicate consisted of material from five plants. (**D**) Increased rate of plant height and root. The values were calculated by the formula: 100·(V_20_ − V_10_)/V_20_, V_10_ and V_20_ denote the value of plant height or root length after 10 and 20 days of Cd treatment, respectively. (**E**), (**F**) and (**G**) Changes of ABA content in new leaf, old leaf and root, respectively. Data are means ± SD of three biological replicates. Each replicate consisted of material from ten plants. *, ** and ***denote statistically significant differences from the control (0 mg L^−1^) at *P* < 0.05, *P* < 0.01 and *P* < 0.001, respectively.

In order to study the response mode of ABA content in ramie plants to Cd stress, we performed the Cd treatment experiment with different Cd concentrations using ramie hydroponic cuttings (Fig. 1). The Cd content of aboveground plants increased with the increase of Cd dose (Fig. 1B). Under Cd stress, leaves of ramie turned yellow gradually, and it became more and more yellow with the increase of Cd concentration (Fig. 1A). The chlorophyll content accordingly decreased: contents of chlorophyll b and total chlorophyll were significantly decreased due to the Cd treatment (Fig. 1C), while the content of chlorophyll a was significantly reduced only on high concentration of Cd stress (30 mg L^−1^). When the treatment concentration of Cd was less than 10 mg L^−1^, the growth rate of plant height and root length of ramie was higher than that of the control, whereas when the Cd concentration was more than 15 mg L^−1^, it was significantly lower than that of the control (Fig. 1D). These results indicated that low concentration of Cd can promote the growth of ramie plants, while high concentration of Cd can significantly inhibit the growth.

To study the change pattern of ABA content in ramie plants in response to Cd stress, ABA content in new leaves, old leaves and roots was analyzed. Under Cd stress, the ABA content was highest in roots, followed by that in old leaves, and the lowest in new leaves (Fig. 1E-G). The content of ABA in new leaves changed little under different doses of Cd stress and different treatment time (Fig. 1E), indicating that the ability of ABA biosynthesis was the weakest in new leaves of ramie under Cd stress. In old leaves, ABA content was the highest after 3 days of Cd stress, and it was higher at the Cd concentration of 30 mg L^−1^ than that in the other concentrations (Fig. 1F), which suggested that high doses of Cd can promote the biosynthesis of ABA. Under various doses Cd, changes of ABA content in roots were first increased (at the third day of Cd treatment) and then decreased (after 10 days of Cd treatment) (Fig. 1G), suggesting that short time treatment of Cd can promote ABA biosynthesis in roots of ramie. Interestingly, the ABA content in root under the dose of 30 mg L^−1^ Cd treatment was significantly lower than that in the control and the other doses treatments after 20 days, denoting that long-time treatment of high Cd concentration may reduce the ability of ABA biosynthesis.

### Effects of ABA treatment on Cd absorption in ramie

**Figure 2:**
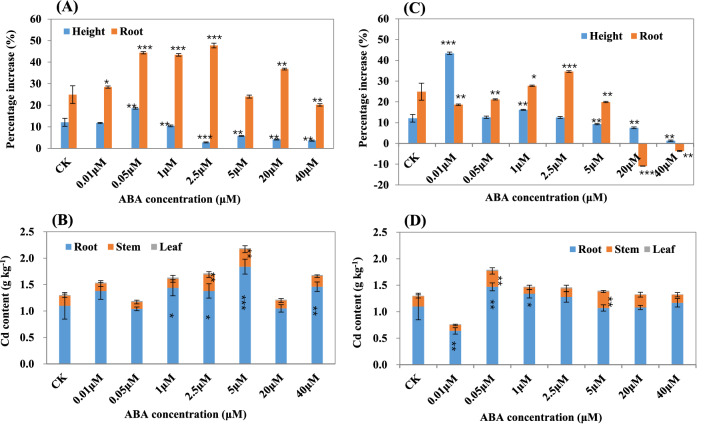
Phenotype and Cd content of ramie plants under ABA treatment. (**A**) and (**C**) Increased rate of plant height and root under ABA treatment by spraying ABA on ramie plants (SORP) and adding ABA directly to the culture solution (ADCS), respectively. Data were calculated by the formula: 100·(A_10_ − A_0_)/A_10_, A_0_ and A_10_ denote the value of plant height or root length after 0 and 10 days of ABA treatment, respectively. (**B**) and (**D**) Cd content of leaf, stem and root under ABA treatment by SORP and ADCS, respectively. Data are means ± SD of three biological replicates. Each replicate consisted of material from ten plants. *, ** and ***denote statistically significant differences from the control (0 mg L^−1^) at *P* < 0.05, *P* < 0.01 and *P* < 0.001, respectively.

In order to study the effects of ABA on Cd absorption in ramie, we performed the ABA treatment experiments by spraying on ramie plants (SORP) or ADCS with different concentrations of ABA, and the Cd content in stems, leaves and roots was analyzed. When the concentration of ABA via SORP was less than 0.05 μm L^−1^, the growth rate of plant height was slightly higher than that of the control, whereas when the concentration of ABA was more than 1 μm L^−1^, it was significantly lower than that of the control, indicating that low concentration of ABA (< 0.05 μm L^−1^) did not inhibit the growth of shoot, but high concentration had the opposite effect (Fig. 2A). When spraying less than 2.5 of ABA μm L^−1^, the growth rate of root was higher than that of control plants, while spraying with 40 μm L^−1^ ABA, it was significantly lower than that of control. The Cd content in root was the highest via SORP, followed with that in stem, and that in leaf was lowest (Fig. 2B). The total Cd content at the concentration of 0.01, 1, 2.5, 5, and 40 μm L^−1^ ABA was significantly higher than that in control, indicating that ABA treatment via SORP can promote the Cd absorption.

ADCS also affected the growth of ramie plants. When the concentration was less than 2.5 μm L^−1^, the growth rate of height was higher than that of control, especially at the concentration of 0.01 μm L^−1^, it was more than three times of control (Fig. 2C). When the concentration of ABA was more than 20 μm L^−1^, roots of ramie decayed gradually and the growth rate of root was negative, suggesting that treatment with high concentration of ABA could lead to root rot, which may cause the slow growth of aboveground plants. Except for the 0.01 μm L^−1^ ABA treatment, the total Cd absorption at the other concentration treatment was higher than that of control (Fig. 2D), indicating that ADCS could increase Cd uptake.

### Expression pattern of chlorophyll-related genes under Cd stress

**Figure 3:**
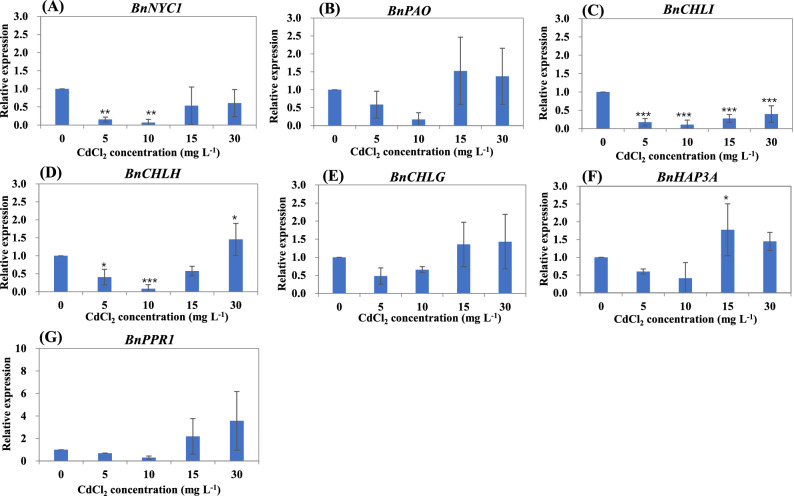
Relative expression of the chlorophyll synthesis related genes (*BnPAO*, *BnNYC1*) and the chlorophyll degradation related genes (*BnCHLH*, *BnCHLI*, *BnCHLG*, *BnHAP3A* and *BnPPR1*) in leaves of ramie under Cd stress. The 2^−ΔΔCt^ method was used to calculate the relative expression of the selected genes. Data are means ± SD of three biological replicates. Each replicate consisted of material from ten plants. *, ** and ***denote statistically significant differences from the control (0 mg L^−1^) at *P* < 0.05, *P* < 0.01 and *P* < 0.001, respectively.

To study the molecular mechanism of the decrease in chlorophyll content, the chlorophyll synthesis related genes (*PAO*, *NYC1*) and the chlorophyll degradation related genes (*CHLH*, *CHLI*, *CHLG*, *HAP3A* and *PPR1*) were chose to analyzed the expression. The homologous gene sequences in rice were used to blast the ramie genome, and the corresponding gene in ramie was named *BnPAO*, *BnNYC1*, *BnCHLH*, *BnCHLI*, *BnCHLG*, *BnHAP3A* and *BnPPR1*, respectively. The expression of *BnPAO* was inhibited under each Cd concentration treatment (Fig. 3A), while the expression of *BnNYC1* was reduced at 5 and 10 μm L^−1^ of Cd treatment but induced at 15 and 30 μm L^−1^ of Cd treatment (Fig. 3B). *BnCHLI* was significantly inhibited under Cd treatment (Fig. 3C), whereas *BnCHLH* was also inhibited by Cd stress except at the 30 μm L^−1^ of Cd treatment (Fig. 3D). Under Cd stress, the expression pattern of *BnCHLG*, *BnHAP3A* and *BnPPR1* was similar, which was reduced at 5 and 10 μm L^−1^ of Cd treatment but induced at 15 and 30 μm L^−1^ of Cd treatment (Fig. 3E-G).

### Expression pattern of ABA synthesis and metabolism genes under Cd stress

**Figure 4:**
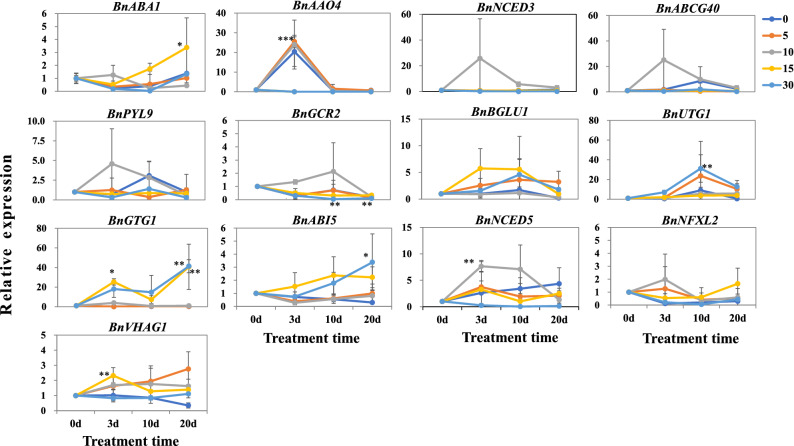
Relative expression of the ABA synthesis related genes (*BnABA1*, *BnNCED3*, *BnNCED5* and *BnAAO4*) and the genes (*BnABCG40*, *BnNFXL2*, *BnPYL9*, *BnGCR2*, *BnGTG1*, *BnBGLU1*, *BnUTG1*, *BnVHAG1* and *BnABI5*) encoding ABA transport and response proteins in leaves of ramie under Cd stress. The 2^−ΔΔCt^ method was used to calculate the relative expression of the selected genes. Data are means ± SD of three biological replicates. Each replicate consisted of material from ten plants. 0, 5, 10, 15 and 30 denote the CdCl_2_ concentration (mg L^−1^). *, ** and ***denote statistically significant differences from the control (samples collected before Cd treatment, 0 d) at *P* < 0.05, *P* < 0.01 and *P* < 0.001, respectively.

To study the molecular mechanism of the ABA content change in ramie plants under Cd stress, the ABA synthesis related genes (*BnABA1*, *BnNCED3*, *BnNCED5* and *BnAAO4*) and the genes (*BnABCG40*, *BnNFXL2*, *BnPYL9*, *BnGCR2*, *BnGTG1*, *BnBGLU1*, *BnUTG1*, *BnVHAG1* and *BnABI5*) encoding ABA transport and response proteins were chose to analyzed the expression. *BnABA1* was up-regulated at the concentration of 15 mg L^−1^ CdCl_2_ for long time treatment (≥ 10 d), but down-regulated in other treatments for different times (Fig. 4). The expression of *BnAAO4* was induced by low concentration of CdCl_2_ treatment (≤ 10 mg L^−1^) for short time (3 d), whereas it was reduced by high concentration of CdCl_2_ treatment (≥ 15 mg L^−1^) for long time (≥ 10 d), which was consistent with the elevated ABA content under Cd stress. When the Cd concentration was 10 mg L^−1^, the expression of *BnNCED3*, *BnABCG40*, *BnPYL9* and *BnGCR2* was high than that of control, and the highest expression was at 3 d excepting the expression of *Bn GCR2*, which showed a peak expression at 10 d. *BnBGLU1*and *BnUTG1* were up-regulated by Cd treatment, and the highest expression was at 10 d. High concentration CdCl_2_ treatment (≥ 15 mg L^−1^) for different times induced the expression levels of *BnGTG1* and *BnABI5*, but decreased the expression abundance of *BnNCED5*. When treated with low concentration of CdCl_2_ (≤ 10 mg L^−1^), the expression of *BnNFXL2* was induced at 3 d, whereas it was reduced at high concentration treatment. When the Cd concentration was lower than 15 mg L^−1^, the expression of *BnVHAG1* was significantly induced, whereas it was inhibited at 30 mg L^−1^ of CdCl_2_ treatment. These results suggested that the changes of ABA content in ramie plants induced by Cd stress may be caused by the differential expression of the ABA biosynthesis, transport and response related genes.

### Expression pattern of ABA synthesis and metabolism genes under ABA treatment

**Figure 5:**
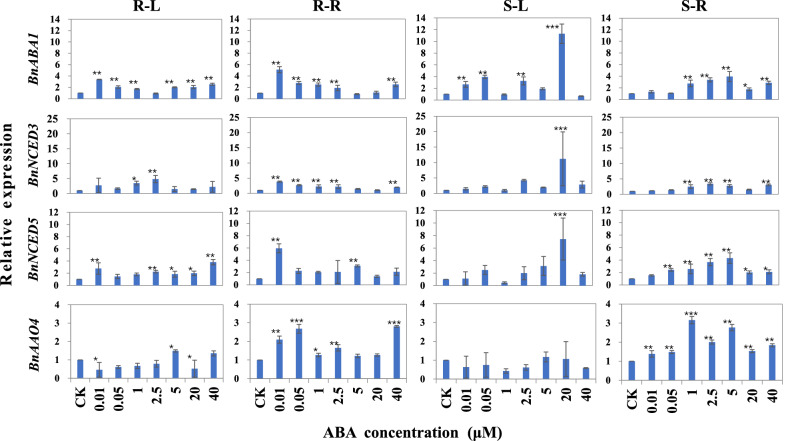
Relative expression of the ABA synthesis related genes (*BnABA1*, *BnNCED3*, *BnNCED5* and *BnAAO4*) of ramie under ABA treatment. S-L and S-R denote the expression in leaves and roots by spraying ABA on ramie plants, respectively. R-L and R-R denote the expression in leaves and roots by adding ABA directly to the culture solution, respectively. The 2^−ΔΔCt^ method was used to calculate the relative expression of the selected genes. Data are means ± SD of three biological replicates. Each replicate consisted of material from ten plants. *, ** and ***denote statistically significant differences from the control (CK, 0 μM) at *P* < 0.05, *P* < 0.01 and *P* < 0.001, respectively.

**Figure 6:**
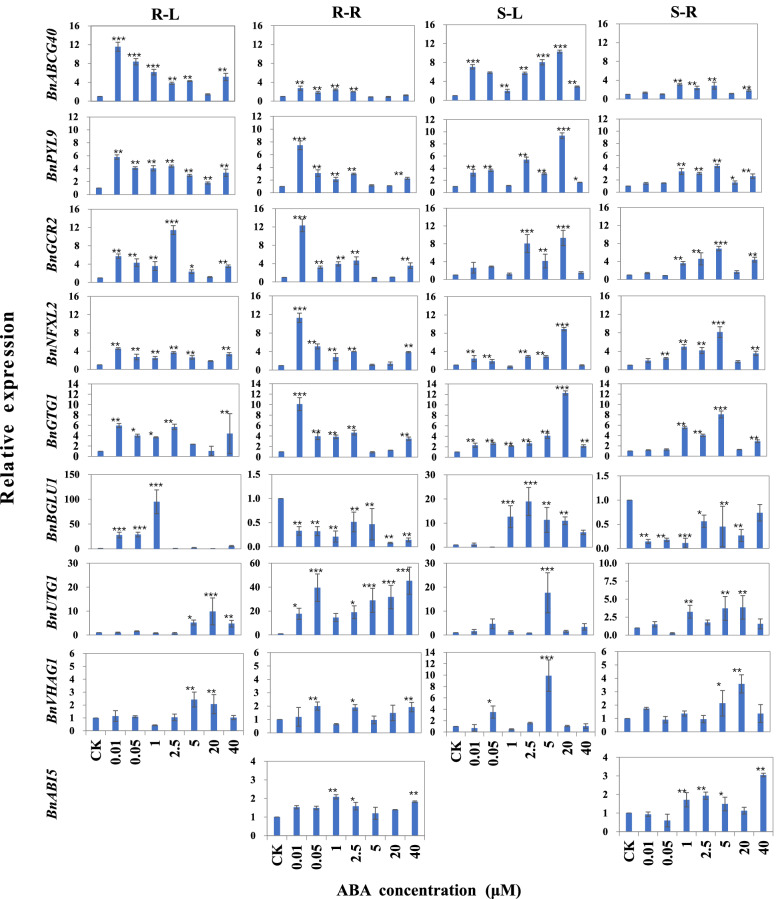
Relative expression of the genes (*BnABCG40*, *BnNFXL2*, *BnPYL9*, *BnGCR2*, *BnGTG1*, *BnBGLU1*, *BnUTG1*, *BnVHAG1* and *BnABI5*) encoding ABA transport and response proteins of ramie under ABA treatment. S-L and S-R denote the expression in leaves and roots by spraying ABA on ramie plants, respectively. R-L and R-R denote the expression in leaves and roots by adding ABA directly to the culture solution, respectively. The 2^−ΔΔCt^ method was used to calculate the relative expression of the selected genes. Data are means ± SD of three biological replicates. Each replicate consisted of material from ten plants. *, ** and ***denote statistically significant differences from the control (CK, 0 μM) at *P* < 0.05, *P* < 0.01 and *P* < 0.001, respectively.

To study the expression patterns of *BnABA1*, *BnNCED3*, *BnNCED5*, *BnAAO4*, *BnABCG40*, *BnNFXL2*, *BnPYL9*, *BnGCR2*, *BnGTG1*, *BnBGLU1*, *BnUTG1*, *BnVHAG1* and *BnABI5*, ABA treatment experiments were performed and the relative expression was analyzed. The expression of *BnABA1*, *BnNCED3* and *BnNCED5* was significantly induced under ABA treatment either by SORP or ADCS (Fig. 5), denoting that ABA treatment may promote the synthesis of endogenous ABA in ramie plants. At the concentration of 20 μM ABA by SORP, the expression of *BnABA1*, *BnNCED3* and *BnNCED5* was sharply accumulated comparing with the other treatment, denoting its leading role respond to ABA treatment. Under ABA treatment, *BnAAO4* was significantly up-regulated in root but down-regulated in leaf, suggesting that *BnAAO4* plays a major role in ABA synthesis in ramie roots. When treated with ABA, the expression of *BnABCG40* and *BnPYL9* was induced compared with control, and it was higher in leaves than that in roots, indicating that they play a dominant role in ABA synthesis (Fig. 6). Under low concentration of ABA treatment (≤ 2.5 μM) by ADCS, the expression level of *BnNFXL2*, *BnGCR2* and *BnGTG1* in both leaves and roots was higher than that under high concentration of ABA treatment (≥ 5 μM). When treated ABA by SORP, the expression of *BnNFXL2*, *BnGCR2* and *BnGTG1* at high concentration treatments (≥ 5 μM) was higher than that at low concentration treatments (Fig. 6). The expression level of *BnBGLU1* in roots was significantly reduced when treated with ABA by SORP and ADCS, but it was largely up-regulated in leaves when treated with < 0.05 μM ABA by ADCS and > 1 μM ABA by SORP, denoting the tissue specific expression of *BnBGLU1* in response to ABA treatment. When exposing to ABA, *BnUTG1*, *BnVHAG1* and *BnABI5* were induced to up-regulated, and the expression in roots was higher than that in leaves, which suggested the dominant role of these genes in root under ABA treatment. These results indicated that ABA treatment can induce the up-regulation of these genes except the expression of *BnBGLU1* in roots, and the induction effect of directly adding low concentration ABA to the culture solution can achieve the effect of high concentration ABA spraying induction.

## Discussion

ABA, known as ‘stress hormone’, plays an important role in plant resistance to heavy metal stress. Ramie being an ideal plant for heavy metal pollution remediation, the regulatory effect and mode of ABA on ramie response to heavy meatal stress is not clear. Previously, we screened a ramie variety Zhongzhu NO2. with strong Cd tolerance and high Cd uptake through hydroponic experiments (Data unpublished). In this study, Zhongzhu NO2. was used as material to study the response mode of endogenous ABA in ramie plant under different Cd concentration stress, and the change mode of Cd uptake in ramie plant under different ABA concentration treatments was also analyzed.

It is confirmed that endogenous ABA content in plants would change under cadmium stress, resulting in changes of Cd accumulation in plant. In rice, Cd treatment can reduce the endogenous ABA content, but induce the accumulation of Cd^27^. While in potato, short-time treatment with CdCl_2_ elevated the endogenous ABA content^17^. In another study, Hsu and Kao (2003)^21^ showed that the endogenous ABA content was induced by Cd stress in a Cd tolerance rice variety, whereas it was not changed in a Cd sensitive rice variety. These papers suggested that the endogenous ABA content showed various change patterns in different crops or varieties. Ramie, a Cd tolerance and high accumulation crop, the Cd accumulation in roots is usually higher than that in leaves^14,26^. In this study, our data showed that the ABA content was higher in roots than that in leaves, consisting with the results in Cd tolerance rice variety. Moreover, we found that the ABA synthesis related genes (*BnABA1*, *BnNCED3*, *BnNCED5* and *BnAAO4*) and the genes (*BnABCG40*, *BnNFXL2*, *BnPYL9*, *BnGCR2*, *BnGTG1*, *BnBGLU1*, *BnUTG1*, *BnVHAG1* and *BnABI5*) encoding ABA transport and response proteins were differentially expressed in ramie when treated with Cd. Together, these data hinted that Cd tolerance crops show high ability of ABA biosynthesis probably resulted by differential expression of the ABA biosynthesis, transport and response genes, which is benefit for its resistance and absorption to Cd.

It is known that the *NCED3*, *ABI5* and *VHAG1* genes are involved in ABA-mediated Cd accumulation in plants^28–30^. Overexpression of *MhNCED3* (*Malus hupehensis*) in *Arabidopsis* reduces the Cd accumulation in transgenic plants^28^, suggesting a negative role of *MhNCED3* in regulating Cd absorption. Meanwhile, the overexpressed *MhNCED3* apple calli shows a higher endogenous ABA level under Cd stress. Our study showed that the expression of *BnNCED3* was induced by both ABA and Cd, and the Cd content in roots was higher than that in control, which was different with the results found in the overexpressed *MhNCED3 Arabidopsis*. In *Juglans regia*, transcription of *JrVHAG1* was induced by ABA, Cd, or ABA + Cd (ABA plus Cd), and the overexpressed *JrVHAG1 Arabidopsis* plants showed a strong tolerance to Cd stress^29^. In ramie, *BnVHAG1* wa*s* induced by ABA and Cd (≤ 15 mg L^−1^), which is consistent with the result in *Juglans regia*. In *Arabidopsis*, the *AtABI5* gene was up-regulated by Cd stress, thereby reducing Cd accumulation^30^. In this study, more than 15 mg L^−1^ concentration of CdCl_2_ treatment can significantly induce the expression of *BnABI5* accompanying with high ABA content in leaves (Fig. 1F), which denotes a different role of *ABI5* gene in regulating Cd absorption between ramie and *Arabidopsis*.

Spraying ABA (< 20 μM) on ramie plants could promote the growth of root with a highest increase rate at 2.5 μM, while the growth rate of aboveground was higher than control on low concentration of ABA (< 1 μM) treatment. The Cd content was highest when spraying 5 μM of ABA, but the heigh and root increase rate showed lower than that at the other treatments. These data suggested that spraying ABA with concentration less than 2.5 μM on ramie plants was the most suitable dose for promoting the growth of ramie under Cd stress and promoting the Cd accumulation in ramie. ADCS could also elevate the Cd accumulation in ramie, however, the growth of ramie plants was inhibited and root rot occurred when treated with high concentration of ABA, which is not conducive to heavy metal remediation. In solanum, a Cd hyperaccumulator, exogenous ABA treatment could increase Cd content^24^, which is consistent with our result. However, Fan et al.^31^ found that exogenous ABA application can decrease Cd accumulation in *Arabidopsis* plants, suggesting that ABA shows different mechanisms of regulating Cd uptake in various species.

In conclusion, we provided evidence that the elevated accumulation of Cd is correlate with the increased endogenous ABA content, and found that the induced ABA content was correlate with the up-regulated expression of the ABA biosynthesis, transport and response genes. ABA application may be potentially a promising approach for inducing Cd accumulation in ramie.

## Supplementary Information

Supplementary Information 1.

Supplementary Information 2.

## Data Availability

All data presented in this study are provided in the manuscript.
