# Supplementary figures and images for: Regulating role of abscisic acid on cadmium enrichment in ramie (Boehmeria nivea L.)
Source: Sci Rep. 2021 Nov 11;11:22045. doi: 10.1038/s41598-021-00322-6 (PMC8585876; doi:10.1038/s41598-021-00322-6)

**Figure S1: The leaf position collected for q-PCR experiment.**

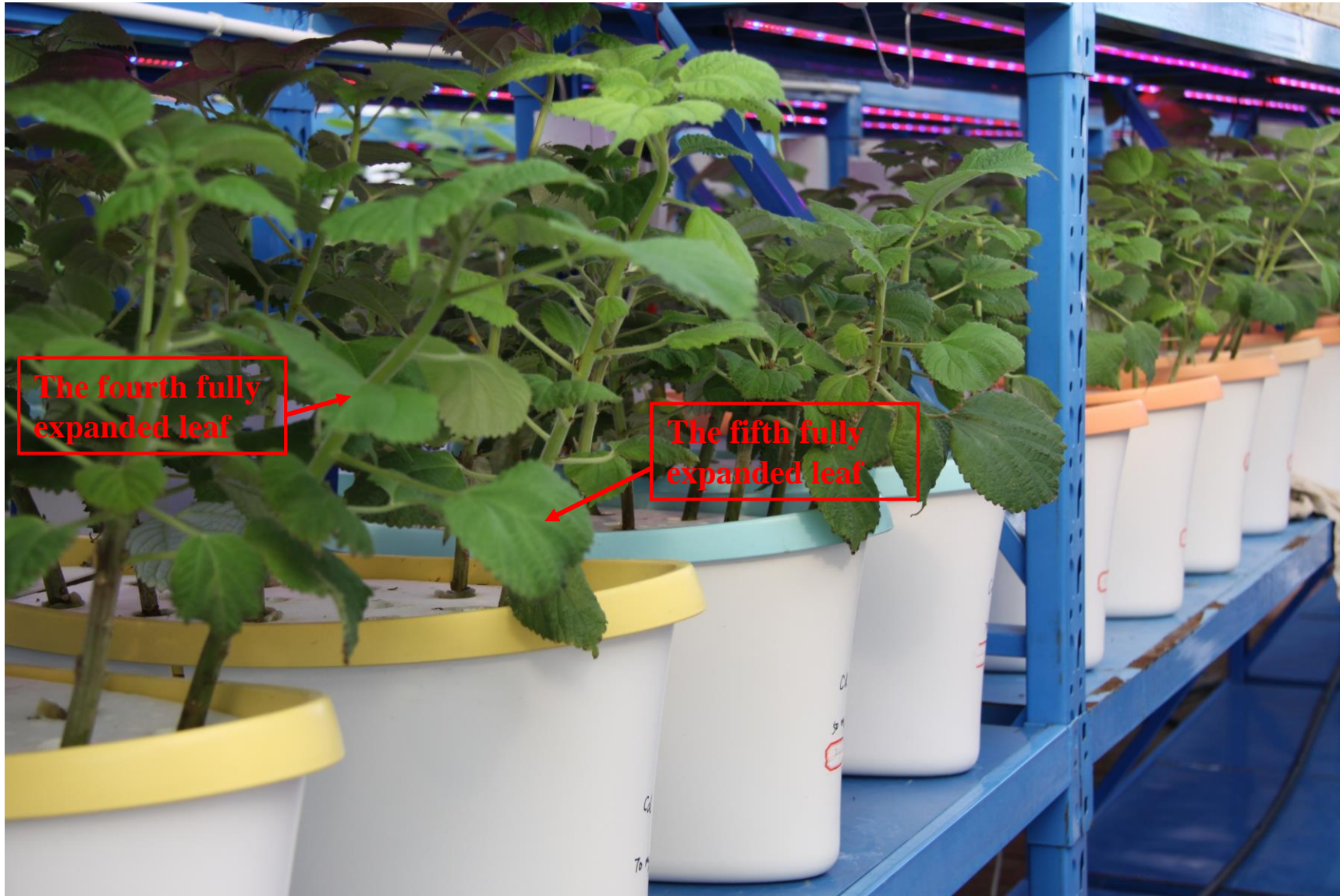

Supplement: Supplementary file 1 — Supplementary Information 1. [file 41598_2021_322_MOESM1_ESM.pdf]
