# Supplementary material for: Regulating role of abscisic acid on cadmium enrichment in ramie (Boehmeria nivea L.)
Source: Sci Rep. 2021 Nov 11;11:22045. doi: 10.1038/s41598-021-00322-6 (PMC8585876; doi:10.1038/s41598-021-00322-6)
Supplement: Supplementary file 2 — Supplementary Information 2. [file 41598_2021_322_MOESM2_ESM.doc]

**Supplemental Tables**

**Supplemental Table.** Primer of the selected genes in q-PCR

| **Gene name** | **Primer-F** | **Primer-R** |
| --- | --- | --- |
| *BnCHLH* | AGCCATGGGTGGACTACTTG | CAGCCACAACAAGCTTCAAA |
| *BnCHLI* | GTGATAGAGGCACTGGGAAATC | ATCTTCAGGGTCCGAGTTGTAA |
| *BnCHLG* | CAATTAGGGCTGCAGAGACTG | CCAAGAAGCTGATTGATGCTC |
| *BnPAO* | GATCTGGGAGACGAAAGCTG | CTCGACCCAGAAGCTGAAAC |
| *BnNYC1* | CAAAAGTCGTTGGCATAGCAT | CCTTTGTTTGTCCCAGCATTA |
| *BnHAP3A* | CAAGGACACCGTCCAAGAGT | ATCGCCATTGATCGTCTTTC |
| *BnPPR1* | TGGAGTTAAGGCCAATCTGTTT | CCTCAGGTTCTGAACTGTACCC |
| *BnABA1* | GGAGCTGATGGTATCTGGTCA | CAATGTCAGCAGGGACAAAAT |
| *BnNCED3* | TCAAACCCCTTCACTCATCC | TGGTTTTGGGGTTTGGACTA |
| *BnNCED5* | GGCTCTCCGGTGATATACGA | CCAGAGGTGGAAGCAGAAAG |
| *BnAAO4* | ATCGATCGGAACCTCCTTCT | CGAAACTCTTGCAAGCATCA |
| *BnABCG40* | TGTTCCGAGGATAAGGGATG | TAAAGGTCCGCAAAATCGAC |
| *BnNFXL2* | CAATAGCATGTCGGAAAAGGA | GCATTGACCAGGATGACAAGT |
| *BnPYL9* | AGTACATTCGGAGGCACCAC | CCAGTGACCATACGAGCTGA |
| *BnGCR2* | GCCTACGTCTTTCTTGCACTCT | TTCCCTTCAGATATGAGCGTTT |
| *BnGTG1* | AGAGTTGGGAGTGGATCTTCAA | TGATGGTCCTGTAATCAGTTGC |
| *BnABI5* | GCAGAGGAGGATGATCAAGAAC | TTTGCTTCCTCTCAAGATCTGC |
| *BnBGLU1* | TCTTGGGCCTTCTTGTTCCT | ACGACTCACAGCTCCTTCAT |
| *BnUTG1* | TGTTCTTCGTCTCTACTCCGG | ATTGGGTGTAGGAGTGGATGG |
| *BnVHAG1* | TCATGGTCCTTTCTGTTCTGGA | ATGCAACAATCTGACCGAAGAG |
